# A SSTDR Methodology, Implementations, and Challenges

**DOI:** 10.3390/s21165268

**Published:** 2021-08-04

**Authors:** Samuel Kingston, Evan Benoit, Ayobami S. Edun, Farhad Elyasichamazkoti, Dawn E. Sweeney, Joel B. Harley, Paul K. Kuhn, Cynthia M. Furse

**Affiliations:** 1 Department of Electrical and Computer Engineering, University of Utah, Salt Lake City, UT 84112, USA; samuel.kingston@utah.edu (S.K.); benej435@gmail.com (E.B.); farhad.elyasichamazkoti@utah.edu (F.E.); sweeneyd20@gmail.com (D.E.S.); paul.kuhn@utah.edu (P.K.K.); 2 Department of Electrical and Computer Engineering, University of Florida, Gainesville, FL 32611, USA; aedun@ufl.edu (A.S.E.); joel.harley@ufl.edu (J.B.H.); 3 Livewire Innovation, Camarillo, CA 93012, USA

**Keywords:** electrical test and measurement, reflectometry, sequence time-domain reflectometry (STDR), spread spectrum time-domain reflectometry (SSTDR), time-domain reflectometry (TDR)

## Abstract

Sequence time-domain reflectometry (STDR) and spread spectrum time-domain reflectometry (SSTDR) detect, locate, and diagnose faults in live (energized) electrical systems. In this paper, we survey the present SSTDR literature for discussions on theory, algorithms used in its analysis, and its more prominent implementations and applications. Our review includes both scientific litera-ture and selected patents. We also discuss future applications of SSTDR.

## 1. Introduction

Reflectometry is a nondestructive technique used to remotely detect, locate, and characterize useful information in physical systems. Reflectometry works by transmitting an electromagnetic (EM) test signal into a system under test, e.g., a transmission line. At each impedance discontinuity within the line, the signal partially reflects and transmits energy. The reflected energy signal returns to a test device for evaluation. The device uses the magnitude and shape of the reflection to determine the impedances in the system. Impedance discontinuities can be located from the velocity of propagation and time delay.

In this paper, we focus on reflectometry for live (energized) systems, with a focus on sequence time domain reflectometry (STDR) and spread spectrum time domain reflectometry (SSTDR) (jointly referred to as SSTDR in this paper). Section 2 covers the basics of reflectometry, including SSTDR. Section 3 discusses its applications. Analysis algorithms are reviewed in Section 4. Section 5 concludes with thoughts on future opportunities and challenges.

## 2. Reflectometry

Reflectometry is a nondestructive technique to remotely detect, locate, and characterize useful information in physical systems. It has been used extensively in electrical systems to locate electrical “opens” or “shorts” [1,2,3,4,5,6], locate cable insulation break down [7], detect electrical system degradation [8,9,10,11], locate ground and arc faults [11,12,13,14], locate damaged components in photovoltaic (PV) systems [12,15,16,17,18], remotely measure complex impedance [17,19,20,21,22], locate intermittent faults that only occur during normal operation [3,4,8,23,24,25,26], and locate faults in three phase systems [27]. In material science, reflectometry has been used to characterize nanostructures, optical fiber networks, waveguides, and dielectrics [28,29,30,31]. Reflectometry has also been used to measure the water moisture content in soils [1,32,33,34]. In this paper, we review reflectometry for fault detection in electrical systems. Reflectometry works by transmitting an EM test wave from a test device through a medium, e.g., a transmission line [3]. At each impedance discontinuity within the medium, the EM wave partially reflects and transmits energy. The reflected EM energy echoes back to the test device for evaluation to produce the reflectometry response. The reflectometry response characteristics and its ability to give the expected information are determined by the type of reflectometry method used [5,6].

### 2.1. Reflectometry Basics

Reflectometry finds faults or other impedance changes within a system by sending an electrical signal (*V_incident_*) into the system and evaluating its reflection (*V_reflected_*) from an impedance discontinuity. There are several types of reflectometry, distinguished by their EM test signal and method of analyzing the reflectometry response [5]. Time-domain reflectometry (TDR) uses either a short rise-time pulse or step voltage signal. Frequency-domain reflectometry (FDR) [35,36] uses a sweep of high-frequency sinusoids. STDR [3], also called binary time-domain reflectometry BTDR) [37], uses a pseudo-random (PN) binary sequence. SSTDR uses a square or sine wave modulated PN code [3]. Noise-domain reflectometry (NDR) [38] uses the native noise present in the system. Chaos-domain reflectometry (CDR) injects true noise signals [39]. Orthogonal multi-tone time-domain reflectometry (OMTDR) enables simultaneous testing and data communication [40,41,42]. Time-frequency domain reflectometry (TFDR) uses a Gaussian enveloped chirp signal as the incident signal and is evaluated in both the time- and frequency-domains [43,44,45]. A full comparison of reflectometry approaches is out of the scope of this review. The relative advantages and disadvantages of the different types of reflectometry depend on the specific system to be tested, its requirements, and details of its optimal implementation. It is important to note that analysis algorithms are often readily transferrable from one reflectometry method to another, and that Fourier transformation and convolution can be used to convert the signatures from one type to those of another. As with all measurement systems, overall performance is limited by bandwidth and sensitivity, which depends on both the hardware and algorithm implementations [5].

The reflected signal carries information on the type and location of the fault. It is controlled by the impedance changes, as measured by the voltage reflection coefficient:

(1) $$\Gamma =\frac{V_{reflected}}{V_{incident}}=\frac{Z_{L}-Z_{O}}{Z_{L}+Z_{O}}$$

where *Z_o_* is the characteristic impedance of the transmission line, and *Z_L_* is the impedance of the load or other impedance discontinuity. The larger the impedance change between *Zo* and *Z_L_*, the larger the reflected voltage and hence, the reflection coefficient. Open (*Z_L_* = ∞) and short circuits (*Z_L_* = 0) produce the largest reflections (Г = 1 and −1, respectively). These large impedance changes are often referred to as “hard faults”. Smaller impedance changes cause smaller reflection coefficients and are sometimes referred to as “soft faults”. Soft faults are more difficult to detect and locate than hard faults. The reflection coefficient is defined in the frequency domain, which means that it can vary with frequency if either *Zo* or *Z_L_* are frequency-dependent (dispersive). Open, short, and resistive loads are not frequency-dependent, so if Г is measured at the load, it is not frequency dependent. Capacitive or inductive loads, or loads connected to transmission lines (where the electrical length varies with frequency) are frequency-dependent. If Г is frequency-dependent, reflectometry analysis often requires conversion between the time and frequency domains.

The distance to the fault is found by multiplying the time delay between the incident and reflected signals by the velocity of propagation (*VOP* is typically about 2/3 the speed of light for most wire types). This means that both the *VOP* and *Z_o_* must be well-known in order to properly locate and diagnose the fault. These values depend on the transmission line, and if the line is not shielded, its nearby environment.

Since each form of reflectometry uses a different incident signal, they will interact differently with a system under test. If a system is live (energized), it is critical that the reflectometry signals do not interfere with the system and vice versa. Interference can be minimized by time-gating the signal (such as for TDR), choosing test frequencies that are out of band from the system (such as for FDR), designing the test signal to be orthogonal to the signals in the system (as in STDR, SSTDR), or using native signals as the test signals (as in NDR).

### 2.2. Sequence Time Domain Reflectometry (STDR) and Spread Spectrum Time-Domain Reflectometry (SSTDR)

**Figure 1:**
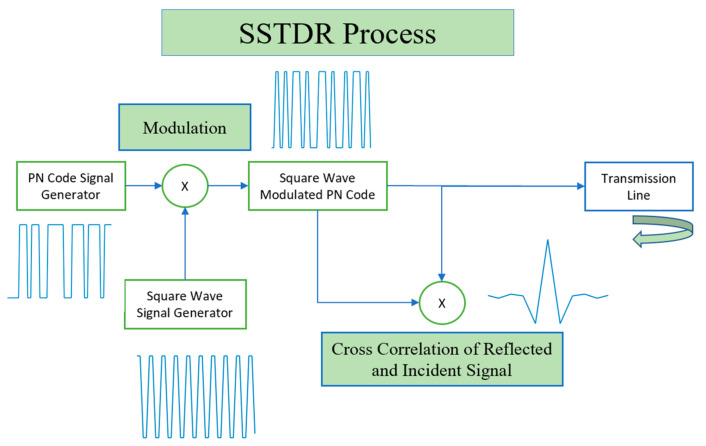
SSTDR process for incident signal, reflection, and cross correlation.

**Figure 2:**
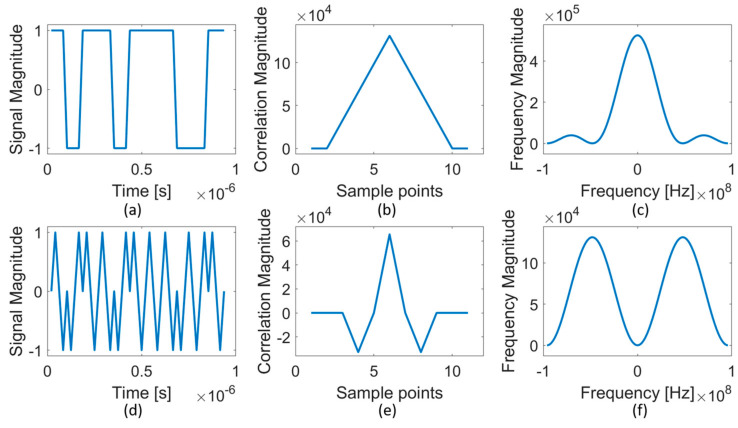
STDR (**a**–**c**) and SSTDR (**d**–**f**) signals. The incident signals (a portion of which are shown) are (**a**) STDR PN code and (**d**) SSTDR sine wave modulated PN code at 48 MHz. The resultant correlation is (**b**) STDR a triangle and (**e**) SSTDR triangle multiplied by a *sinc*. The frequency spectrum of the correlated signals in (**b**,**e**) are shown in (**c**,**f**).

The SSTDR process is shown in Figure 1, and the detailed signals are shown in Figure 2. In STDR, the test signal is made up of a PN binary code [3,46], shown in Figure 2a. The incident PN code is launched from the test device and experiences partial reflection and transmission at each impedance discontinuity in the system under test. The reflections that return to the test point are cross-correlated with a delayed copy of the incident PN code to produce the STDR response (Figure 2b). SSTDR uses a square- or *sine*-wave modulated PN code as the test signal (Figure 2d) and produces a *sine*-like correlated reflection signature (Figure 2e). If the system is frequency-dependent or lossy, the reflected signal will experience dispersion and attenuation, and the shape and magnitude of the reflectometry response will be different.

SSTDR has some distinct advantages. It can be used on live electrical systems with minimal interference with signals already present in the system [47]. It has natural noise immunity [5] so experiences minimal interference from existing signals in the system or external electrical noise sources. It possesses a dynamic frequency-domain bandwidth that can be varied by the modulation frequency [6]. The ability to send signals on energized systems without interfering with them comes from making the amplitude of the PN code (Figure 2a,c) very small compared to the native signal in the system under test. The SSTDR signal can still be received, using correlation over time. This correlation gives it immunity from noise and other existing signals in the system. The cross correlation between the SSTDR signal and these noise/existing signals will be very small, while the cross correlation with the SSTDR signal will be large. This allows extraction of the SSTDR response without interfering with/being interfered with by noise or existing signals in the system under test. PN codes have another important feature, in that cross correlation between two different PN codes is very small [48]. This depends on the type of code used. Maximum length (ML) codes are best for systems with only one PN code, as they have the best noise immunity. Kasami codes, which have minimal cross correlation between codes, are a better choice when multiple codes are used, such as in networks where multiple SSTDR units might be needed or cross-coupling may occur between unshielded wires. The ability to use multiple non-interfering codes on the system enables parallel simultaneous measurements. There are numerous applications today where a vector network analyzer (VNA) is connected to a large switching network to measure transmission and reflection between multiple antennas. This is seen, for example, in medical imaging (magnetic resonance imaging (MRI), microwave imaging, etc.), where each antenna transmits sequentially, and its reception is measured at every other antenna in turn. Being able to measure the reflection and transmission between every antenna and every other antenna could be done in parallel with multiple SSTDR circuits simultaneously transmitting and receiving different PN codes. Each SSTDR would transmit a different PN code, which would reach every other antenna. If each SSTDR had a set of correlation circuits working in parallel, it could simultaneously receive and measure the response from every other antenna. This would enable nearly instantaneous medical imaging. Since the cost of a medical procedure is often billed by the minute, this reduces the cost substantially. It could also enable functional imaging of moving or time-varying structures in the body (beating heart, etc.). In addition, this method would remove the need for a high-precision switching network, which is often the limiting factor for hardware sensitivity.

This new application will require circuitry that also enables measuring transmissions in addition to reflections. The savings in time that would be expected to result from this parallel testing could potentially enable medical applications that are not feasible today, including the testing of functional biological activities that vary quickly with time.

The frequency spectrum of the SSTDR signature enables it to function on live systems. A segment of a PN code used in the STDR system is shown in Figure 2a and for SSTDR in Figure 2d. When correlated, these signals provide the time domain signatures for STDR (Figure 2b) and SSTDR (Figure 2e). The Fourier transforms of the STDR and SSTDR signatures are shown in Figure 2c,f, respectively. This indicates the level of energy in each frequency across the spread spectrum. STDR is centered at DC, with nulls at the primary frequencies of the PN code. SSTDR shifts the STDR spectrum to center it at the modulation frequency of the PN code. Evaluating where the SSTDR signature overlaps the spectrum of existing signals and noise is important for optimizing the design of the SSTDR system for energized systems. Lower frequencies, for example, propagate better in lossy (attenuating) environments (where STDR excels), and higher frequencies provide higher resolution (where SSTDR, particularly those with higher PN code frequencies, has an edge). The bands can also be chosen to optimize regions where measurement is most desired (such as frequency ranges of impedance measurement) and to avoid regions where noise margins are tight. Testing with both STDR and SSTDR in parallel (using different PN codes for each), and potentially using multiple instances of SSTDR with different modulation frequencies could be used to improve the testing bandwidth in future systems.

A primary benefit of SSTDR comes from its ability to monitor live electrical systems, which allows it to be installed alongside an existing system and remain in-place to perform system health monitoring for the life of the system. The first SSTDR implementation utilized an analog correlator, which obviated the need for fast sampling [3]. The versatile FPGA has been used in more recent systems to implement a digital SSTDR [49]. Multiple application specific circuits for SSTDR have been designed to meet the various needs of electrical systems [50,51,52,53]. CMOS mixer circuitry using MOSFETs has been designed for SSTDR implementations with the intent to reduce total harmonic signal distortion and lower power consumption [54,55]. In another implementation for aircraft wiring, the correlation component was implemented by an acoustic-optical crystal to improve the fault detection resolution [56].

SSTDR has been applied in a variety of situations to monitor electrical system health and to both detect and locate faults. It has been used in airplane cabling where it has been able to detect intermittent faults lasting a few milliseconds [47]. Application has also been seen in power systems [27,57,58], PV systems [11,12,13,15,16,18,59,60], undersea cabling [61], and railroad systems [49,62,63]. It has been proposed that SSTDR could be used in battery health monitoring [64] and cable degradation monitoring [65].

### 2.3. Reflectometry Challenges

All reflectometry systems have some interesting challenges, which we will briefly describe in this section.

#### 2.3.1. Multiple Reflections

Since reflections occur at every impedance discontinuity, multiple overlapping reflections often occur. For instance, when a signal is sent from the reflectometer into a transmission line, a first reflection is typically seen at this location. The signal then travels down the transmission line to the load, where it reflects and returns to the reflectometer. It is also again reflected at the junction between the line and reflectometer, again traveling to the load and back. All of these reflections will be seen in the reflection signature, and often they will overlap. This is important to consider when developing algorithms for evaluating the reflectometry response, which are described in Section 4.1.

Multiple reflections also create a “blind spot” [66] near the junction between the reflectometer and cable, where smaller faults near the start of the cable are “blinded” by the large initial reflection. This can be eliminated by subtracting a baseline (good) measurement from measurements of faulted data. When a system has multiple paths for current, such as branched networks, the multiple reflection problem becomes even more significant, because reflections and transmissions occur at each branch. As the signal propagates, each reflection becomes smaller and smaller, making it progressively more difficult to identify and locate faults that occur several junctions from the reflectometer.

#### 2.3.2. Small Reflections

The reflection coefficient in (1) represents the ratio of the reflected to incident voltage. When the impedance discontinuity is small (a “soft fault”), the reflected voltage is also small. Several specific algorithms have been developed for locating small faults, as described in Section 4.1. The ability to locate soft faults is limited by the noise in the measurement system (which can be reduced through averaging and other de-noising techniques), and by normal impedance variation in the system [7]. Soft fault detection and location remains an area of considerable interest. Future research may make use of “incipient fault” detection methods from other applications such as control and process engineering [67,68].

#### 2.3.3. Intermittent Faults

Many faults occur due to intermittent causes such as water ingress or vibration. These faults may last a very short time, but later turn into more serious, permanent faults, similar to the incipient faults mentioned above. Arc faults are intermittent short circuits that cause unplanned surges of current. They are often implicated in fires and damage to the system [69,70]. Arc faults may be series or parallel, and their duration, magnitude, and details depend on the power/signals on the line, the length and type of wires and connections, the loads, and the protection systems [71]. Higher power tends to reduce the duration arc faults, as the wires or other connections burn away more quickly. Wires that are larger and more robust tend to increase the duration of arc faults. Wet arcs (such as may be caused by water bridging cables with damaged insulation) tend to burn away very quickly (<1 ms) as current heats the water. Dry arcs (such as may be caused by vibration of chafed wire against nearby metallic parts) tend to last longer, as they are caused by mechanical vibration on the order of seconds. Thus, faults may be a fraction of a millisecond in duration to a few seconds. The longer a fault lasts and the larger power it supports, the more heat is generated, and the larger its chance of causing a fire or other damage. Methods for reducing the hazard due to arc faults such as arc fault circuit interrupters [72] and rapid shutdown systems [73] most often focus on detecting the intermittent changes in voltage and/or current and shutting the system down quickly. Location of the fault is generally not available with these methods, thus impeding the maintenance inspections that follow.

Reflectometry methods have been used for locating intermittent faults. These include TDR [74,75], multicarrier reflectometry [76,77], SSTDR [47,78], and CDR [79]. Locating intermittent faults means that the measurement system must be active at the time the fault occurs and be able to complete the test in the length of time the fault is present. If the test is taken while the fault is time-varying, this can cause significant degradation to the reflectometry response. Thus, it is useful to make multiple reflectometry measurements during the duration of the intermittent fault. This requires a short duration reflectometry signal and fast analysis. The ability to improve the signal using averaging is therefore limited.

## 3. SSTDR Applications

With a growing need for an effective diagnostic system that can keep critical systems functioning, great interest has been shown in SSTDR. This method is fast becoming a favored technique for monitoring the state of health of many electrical systems. The following is a survey of the various implementations and applications found in the literature.

### 3.1. Diagnostics for Aircraft Wiring Systems

SSTDR was originally designed for locating faults on aging aircraft cables [3]. These faults are particularly challenging, because they are often intermittent [80], caused by heat, vibration, water ingress, mechanical or electrical stress, etc. Such faults can cause arcing and lead to fires. As described above, dry arcs are caused by mechanical vibration or other problems that lead to wires making direct contact with each other or nearby ground structures. Wet arcs are caused by water bridging damaged or aged insulation. Dry arcs tend to last a fraction of a second whereas wet arcs are on the order of milliseconds. Both can be detected and located by SSTDR [47,78]. SSTDR’s ability to test energized systems has been demonstrated for 440 Hz aircraft wiring [47], wires carrying power line communication signals [81], Mil-Standard 1553 data signals [3,82], and ARINC825 signals [83]. It has been considered for both diagnostics and prognostics [84], and has been used on both airplanes and helicopters [85,86].

### 3.2. Transmission Lines and Cabling

SSTDR instrumentation has been applied to transmission line and cabling evaluation, for example on digital subscriber line (DSL) [87], power cables [1], aging nuclear cables [65,88], cables used to support subsea oil and gas drilling [89], and control cables in rail systems [62,63,90]. It has also been shown that the SSTDR technique can be coupled to transmission lines using non-contact coupling approaches [91]. Combining SSTDR with GPS data can enable geographic identification of fault location [92], or combining it with design data can map fault location to specific locations within an aircraft [93].

### 3.3. Motor Vehicles and Batteries

With increasing complexity in the electrical systems, SSTDR can be applied to the diagnostics of automobile wiring networks [94]. This can be extended to buses, trucks, and many other motorized vehicles. Many of these vehicles use a controller area network (CAN bus) for control and instrumentation [94]. SSTDR could be used to find faults in the wiring or in the batteries (e.g., Li-ion cells), where impedance changes are early indicators of state of health.

### 3.4. Power Systems

SSTDR has been used to determine the quality of devices used in power systems, where change in impedance may indicate a change in health. SSTDR has been used to monitor the status of power converters [95,96], power-switching devices [27,58,97,98], IGBT power modules [9,98], IGBT bond-wires buried cables [99], and MOSFETs [10,100,101,102]. For example, ref. [8] used SSTDR to track real-time degradation of energized MOSFETs that were being thermally aged in an environmental chamber, while ref. [103] also showed that SSTDR could be used to monitor impedance changes and determine the amount of degradation a thermally-aged MOSFET has suffered. Knowing these changes, one could also estimate the remaining lifespan of the MOSFET. SSTDR has also been used to identify bond-wire faults in aged and fatigued IGBTs and MOSFET circuits [101]. Power switches in power inverters also age and degrade with power cycling. SSTDR techniques have been shown to successfully characterize the aging of power-switching devices by monitoring changes in the device’s impedance. For example, SSTDR signals were embedded within the gate driver signals and used to characterize power-switch impedances [27].

New techniques to reduce excess diagnostic data and ease SSTDR’s implementation are being actively developed to improve state-of-health monitoring [57,97]. One of the challenges of using reflectometry on energized systems is to provide sufficient circuit protection for the SSTDR hardware. One way to do this is to use SSTDR to test while the system is at, or near, its zero-crossing state [104].

### 3.5. Photovoltaic Systems

An overview of using SSTDR with PV systems was presented in [11]. SSTDR has been used on live PV arrays to find the position of wiring faults [105], ground faults [13,106], arc faults [12], disconnection faults [107], accelerated degradation [11], shading faults and broken panels [60,108]. The feasibility of using SSTDR to detect and locate faults for PV is summarized in Table 1.

### 3.6. Measurement of Complex Impedance

Electrical impedance is a fundamental electrical measurement. It is used to evaluate all types of electrical circuits and systems, composition and moisture in foods, materials for manufacturing, building materials, soils and agricultural products, and more. At high frequencies (kHz to GHz ranges and up) the VNA is the most common tool for impedance measurement. A VNA can be thought of as a type of frequency domain reflectometer. It sends a set of sequential sinusoidal signals into an electrical system (often using a frequency chirp pulse). The magnitude and phase of the reflected signals are measured, giving the complex reflection coefficient (S_11_ = Г). Impedance is then calculated from (1). If the VNA has multiple ports, both the complex reflection and transmission coefficients are measured. Using the Fourier transform, time domain reflectometry methods can also be used to measure reflection, and hence impedance, similar to the VNA. TDR is regularly used for measuring soil moisture, and preliminary results have indicated the potential of SSTDR for measuring complex impedance [15,19,20,107,108,109].

When reflectometry (including SSTDR) is used to measure frequency-independent loads (open or short circuits or resistive loads), the reflection that is returned is a symmetric, scaled version of an open circuit reflection. The scaling factor is dependent on the reflection coefficient. However, many loads are complex and frequency-dependent (dispersive). In these cases, the time-domain reflection response changes both magnitude and shape. These shapes provide information that represents the complex impedance, such as capacitance and inductance [22]. Advanced algorithms such as machine learning and dictionary matching techniques have been used to identify the complex loads [18,19,110].

In future applications, the ability of SSTDR to measure complex impedance, combined with its ability to test on energized systems, natural noise immunity, and ability to make multiple measurements in parallel using different PN codes, could enable applications that are difficult or impossible today. Today’s SSTDRs are designed for measuring reflection but incorporating methods to enable them to also test transmission between SSTDR units could make them a viable replacement for the VNA. This would create an SSTDR-VNA that could test on live systems, systems in electrically noisy environments, or systems (such as medical imaging) where parallel testing is advantageous.

### 3.7. Tamper Detection

Power systems are often subject to cable theft or vandalism, leading to the need for tamper detection capabilities. SSTDR has been shown to be one of the options for the detection of tampering in nuclear power [111] and cable theft in rail applications [62].

### 3.8. Structural Health Monitoring and Small Impedance Changes

SSTDR has been used for structural health monitoring of pre-stressed concrete stranded cables in dams [112]. These anchors must be electrically isolated from other parts of the structure, which limits the use of reflectometry in structural health monitoring. This application is particularly significant, however, because the breaks in these stranded cables produce reflections that are very small (on the order of one millionth of the reflection from an open circuit). In order to locate these very small faults, the signal-to-noise ratio of the measurement system needs to be enhanced. This was done by averaging the responses for a long time (9 s, in this case). Averaging the SSTDR signature is carried out after the correlation. Using either longer PN codes or averaging multiple SSTDR signatures can both reduce the measurement noise in the system, making it possible to find smaller and smaller reflections reliably.

Baselining [7,113] was also used in this application. This is commonly used to enhance the ability of SSTDR to locate small changes in impedance. A baseline is taken when the system is considered “good”. The baseline includes the effects of reflections from all normal impedance mismatches in the system (such as cables, junctions, loads, etc.). The baseline is then subtracted from each subsequent measurement to remove the reflections from these normal changes. When a change in impedance occurs, the change is the only reflection remaining after baseline subtraction, thus augmenting this change and making it easier to detect and locate.

It is important to recognize that SSTDR is measuring impedance changes, and that any change that occurs in the normal operation of the system will be detected right along with all unexpected changes from faults. Normal changes can be caused by switching or impedance changes in loads, irradiance levels, rain and humidity changes, vibration [84] acceptable moisture ingress, and more. If these normal impedance changes are larger than the impedance changes from faults, they will mask the faults and make them invisible [7]. This variability has been measured for aircraft cable fault location [7,114] and PV systems [115]. Averaging multiple SSTDR signatures is particularly helpful when there are random impedance variations in the system. This can remove random impedance noise, while leaving the non-random changes that would indicate a fault.

## 4. SSTDR Signal Processing Algorithms

The SSTDR reflection signature contains information about the type and location of impedance mismatches within the system. To identify and locate these changes, signal processing algorithms are used. This is one of the areas of SSTDR research that still has many untapped opportunities for improving the application-specific capabilities, accuracy, and efficiency of these algorithms. Examples of existing algorithms for SSTDR evaluation, methods to predict how well they will work, and methods to improve their accuracy are described in this section.

### 4.1. Inverse Methods

Inverse methods are used to evaluate the SSTDR reflection responses and infer the state of health of the system (including location of faults or impedance changes). One class of methods uses sample measurements (or simulations) of a system in various states (with and without faults in various locations) to create a “dictionary”. This dictionary is then compared to measured data to determine the health of the system and location(s) of faults. The dictionary matching methods has been used to measure the capacitance of an end-terminated load [19] and disconnection fault location in a PV system [18].

Classical steepest descent inverse algorithms have also been applied to SSTDR data [20] to evaluate series connected resistance-capacitance (RC) loads. Steepest descent iteratively identifies loads that minimize the misfit function by comparing simulated model data (SSTDR time-domain reflection signatures) to measured data.

Machine learning algorithms, including the continuous wavelet transform and probabilistic neural networks have been implemented for SSTDR [82]. Radar imaging methods [116] and numerous algorithms are used in other types of reflectometry analysis and could be applied to SSTDR. These include electromagnetic time-reversal (TR) [117,118], decomposition of the time reversal operator (DORT) [119], and time-reversal-based multiple signal classification (TR-MUSIC) [120]. For soft faults, algorithms include a matched-pulse approach in [121], matched filter [122], sliding correlators [123,124], inverse scattering [125], using a combination (cluster) of reflectometry measurements plus crosstalk between multiple wires in a bundle [126], an iterative deconvolution [127], and BTDR algorithms to remove large reflections and emphasize small reflections [128]. For branched networks, genetic algorithm (GA) [129,130,131], neural networks (NN) [130], particle swarm optimization [132], teaching–learning-based optimization [133], backtracking search optimization [134], inverse scattering [125], use of symmetry to resolve hidden or overlapping peaks [135], and algorithms that use iterative evaluation of the network [129,136,137] have been developed.

### 4.2. Forward Modeling

In order to analytically evaluate how SSTDR will work in various PV arrangements, simulation engines have been developed to predict the SSTDR response. Three general methods have been used for this analysis—time domain solvers (e.g., the finite-difference time-domain (FDTD) method) [114,138], graph network theory [139], and frequency domain solvers such as the systematic solution procedure (SSP) [16,140]. Full simulations that consider the lengths and impedances of cables and elements within the system have been developed for PV [16,138], multiconductor transmission lines above a ground plane [141], ground faults [14], line-to-line faults, and open-circuit faults. Simulated “digital twins” can be used to evaluate how various analysis algorithms will work or can be used directly within the algorithms themselves (as in dictionary matching).

### 4.3. Improving Accuracy

Averaging and baselining [7,113], described in the previous section, can significantly improve the accuracy of SSTDR algorithms. Averaging is explicitly described for structural health monitoring in ref. [118], for disconnections in PV systems in ref. [117], and for ground faults in PV systems in ref. [13]. Since SSTDR data is sampled in time, the peaks are often missed. If peak detection algorithms are used, this can result in inaccuracy in both the location and magnitude of the impedance change. Methods to fit or extrapolate these peaks [142], or the use of a sign eliminator [143], can significantly improve the results.

## 5. Conclusions and Future Directions

SSTDR provides a test mechanism with three key features. It can be used on live (energized) systems and/or in electrically noisy environments. It can detect extremely small changes in impedance by improving the SNR via averaging, baselining, and additional signal processing. It can test on multiple coupled parts of a system simultaneously by using different PN codes. Applications already demonstrated include detection and location of open and short circuits in aircraft, rail, and cable diagnostics (nuclear, subsea oil and gas, etc.). Emerging applications will expand on this capability and measure complex impedance. A future potential application is replacing the vector network analyzer used today and expanding to impedance measurements on live and time-varying systems. SSTDR could also enable parallel testing in applications that require parallel measurements between multiple antennas, such as medical imaging (magnetic resonance imaging (MRI), microwave imaging, etc.). This new application will require circuitry that also enables measuring transmissions in addition to reflections. The savings in time that would be expected to result from this parallel testing could potentially enable medical applications not feasible today, including the testing of functional biological activities that vary quickly with time.

With both existing and emerging applications, there is a need for algorithms for impedance measurement, fault location, etc., particularly in complex systems (PV arrays, for instance). These algorithms may be more advanced, giving better results, or they may be simpler, thereby enabling faster processing on smaller, less expensive devices. There is also an opportunity for improved SSTDR circuitry that is smaller, faster, more efficient, and less expensive (a new ASIC, for instance). SSTDR is basically a time-domain correlator, and there are numerous ways to do this, with and without the need for fast sampling. Furthermore, opportunities for using multiple PN codes to test in parallel, including the ability to use transmissions and reflections to test a system, open up a demand for improved circuitry. In most applications, the best results are obtained using baselines of the system in its initial state. The baseline is best for an embedded system that is left in place, not disconnected and then reconnected (thus changing—even slightly—the connection impedance). Thus, small, power-efficient, low-cost devices are needed to enable embedded applications.

Finally, each application has different demands in terms of impedance sensitivity for detection, length and loss of cables, signals on the line, and noise tolerance, etc. Fully understanding the need of each application and tailoring the SSTDR approach to that application is important to obtain the best results. For instance, STDR can be used at low frequencies and SSTDR at higher frequencies. Combining both methods, potentially in parallel, can provide the broadest bandwidth for measurement. SSTDR provides numerous opportunities for combining the electromagnetics of reflectometry with the strength of signal processing in the time- and frequency-domains as well as analog and/or digital circuitry for emerging applications in test and measurement.

## Figures and Tables

**Table 1:** Overview of the SSTDR technique for fault detection and location. a “D” or “L” indicate the method has been demonstrated for detection or location, respectively. * means it is feasible that the method could be used, but it has not been demonstrated from [11].

Fault	Detection (D) Location (L) * = Feasible	Ref.
Open/Short Circuit	D, L	[107]
Connection Fault	D, L	[107]
Ground Fault	D, L *	[13]
Arc Fault	D, L *	[12]
Shading Fault	D, L *	[108]
Bypass Diode Fault	D *, L *	
Broken Panel	D *, L *	[60]
Accelerated Degradation	D, L *	[11]

## Data Availability

Not Applicable.
